# A defense of surgical procedures regulation

**DOI:** 10.1007/s11017-022-09569-0

**Published:** 2022-05-12

**Authors:** Mattia Andreoletti, Federico Bina

**Affiliations:** 1 Health Ethics and Policy Lab, Department of Health Sciences and Technology, ETH Zürich, Zürich, Switzerland; 2 Faculty of Philosophy, Vita-Salute San Raffaele University, Milan, Italy; 3 Department of Psychology, Harvard University, Cambridge, Massachusetts United States

**Keywords:** Evidence-based medicine, Health Ethics, Paternalism, Philosophy of Medicine, Sham surgery, Surgical RCTs

## Abstract

Since the advent of drug regulation in 1962, regulatory agencies have been in the practice of using strict standards to test the safety and efficacy of medical treatments and products. Regulatory agencies, such as the FDA, demand two full-fledged Randomized Clinical Trials demonstrating the safety and effectiveness of drugs to grant its marketing authorization. On the contrary, surgical treatments are left completely unregulated. There are several reasons explaining this difference, and all of them point to the difficulty of conducting well-designed RCTs in surgery. However, we argue that none of these arguments is decisive and that, under certain conditions, surgical RCTs can be morally justified and methodologically sound. Although ethical constraints restrict the number of testable surgical procedures, and surgical trials might not be as dependable as pharmaceutical RCTs, our analysis suggests that, in certain cases, it is possible to obtain high-quality evidence about the safety and efficacy of surgical procedures. Untested surgical treatments may prove to be ineffective and harm patients. Therefore, regulation of surgical procedures seems not only morally acceptable and able to provide reliable scientific evidence, but also desirable and justified from an ethical-political standpoint.

## Introduction

Since its founding in 1906, the United States Food and Drug Administration (FDA) has become one of the largest and most influential regulatory agencies. Monitoring a market of products worth over 1 trillion dollars, which represents nearly a fourth of consumers’ spending in the United States, the FDA has paved the way for drug regulation in all western countries. Since the famous Kefauver-Harris Amendment to the Food, Drug, and Cosmetic Act in 1962, pharmaceutical regulatory agencies all over the world have similarly required evidence of a new drug’s safety and efficacy before granting market approval. Concerning pharmaceutical treatments, the FDA demands two full-fledged Randomized Controlled Trials (RCTs) demonstrating the safety and effectiveness of the new drug under testing to grant market approval. RCTs are large experiments involving human participants in which an experimental drug is tested either against the standard of care or a placebo. This experimental design is considered to deliver the most reliable scientific evidence for the safety and efficacy of a drug, therefore allowing regulators to make scientifically sound choices about pre-market approval.

The FDA is generally considered a successful agency^1^ if one considers drug withdrawals as a reliable empirical benchmark [1]. For instance, on average in the US between 1993 and 2006 only 1.5 drugs per year were withdrawn from the market, and the number of withdrawals has not increased over time [2]. Moreover, the agency performed well in protecting patients from unsafe and ineffective products [3].

For many readers of this journal it might be surprising to know that there is no FDA-like authority either directly regulating existing surgical procedures or approving the use of new ones. Surgical procedures are usually transmitted from senior to young surgeons, slightly refined over time. Based on surgeons’ experience, procedures might be abandoned or completely transformed into new techniques, due for instance to technological developments. As a result, surgeons can easily introduce new procedures into practice without the need for testing in clinical trials. So, contrary to pharmaceutical products, so far “the development of surgical procedures has not depended upon the RCT but rather upon an enthusiast performing a case series” [4].

Since the advent of the Evidence-Based Medicine paradigm (EBM), there is a large consensus in the medical community on the rules of evidence: RCTs are widely considered to deliver the best evidence about the safety and efficacy of treatments. This is because, as mentioned above, the experimental design of RCTs allows researchers to minimize biases, therefore providing the strongest evidence for a hypothesis. Other study designs and forms of evidence are instead considered to be less valuable. For instance, case reports and case series, which are often the only evidence base on which surgical procedures are developed and introduced in clinical practice, are at the bottom of the EBM hierarchy of evidence, even lower than experts’ opinion. Indeed, case-based studies are simple, descriptive ones, reporting a new approach or treatment to a particular medical condition, providing, therefore, no robust evidence. And translating results from these studies to general clinical practice without stronger “experimental” evidence might be harmful to patients. But this is how surgical innovation ordinarily works.

As a result, patients can access either pharmacological treatments tested in full-fledged RCTs or surgical procedures, which have not been through the same strict testing process. For example, let us imagine a patient suffering from lower back pain. There are two general treatment options: drugs, like a painkiller or a muscle relaxant, and surgery. In the former option, the patient can access treatments that have been tested in rigorous clinical trials, and for which there is robust evidence for safety and efficacy. In the latter case, the patient can undergo a surgical treatment that has never been tested in RCTs, and whose evidence for safety and efficacy has never been reviewed by any public agency. The existence of such a ‘double standard’ has already been detected, debated, and criticized since the 1970s [5–7]. For instance, Spodick [5] analyzed the reported evidence for medical and surgical treatments for diseases of the heart and great vessels, and he found that for surgical treatments no controlled trials had ever been published. But, intuitively, comparable–though proportionate–evidential standards should be sought, even if only to compare risks and benefits of different potential treatments: from (capable) patients’ side, to make informed therapeutic choices as much as possible free from distorting influences; from the physicians’ side, to provide the most complete picture of the therapeutic alternatives; from health managers’ side, to allocate limited resources cost-effectively.

Nonetheless, as of today, systematic testing of surgical procedures through regulatory policies is out of the discussion, and “many surgeons remain suspicious of this kind of tentative protocol-driven medicine when applied to surgical practice” [8]. There are indeed *epistemological*, *pragmatic*, and *ethical* reasons for the lack of systematic statistical testing of surgical procedures. And all of them point to the difficulty of conducting RCTs in surgery. Below, we review the main arguments against conducting RCTs in surgery, and we show that such arguments can be questioned. In what follows, we show that in surgery at least some approximations to RCTs are both possible and morally desirable if some conditions are met. Finally, we argue that regulation of surgical procedures is justified from an ethical-political standpoint, and we briefly explain how that might work.

## Epistemological and pragmatic concerns in surgical RCTs

On the epistemological side, the main issue is that some key features that render pharmaceutical RCTs reliable sources of evidence cannot be implemented in surgical trials. Two key methodological devices seem particularly problematic to implement for testing surgical interventions: the standardization of the procedure, and blinding.

### The standardization of the procedure

First, surgical treatments are often difficult to standardize in the way that RCTs require. Surgical interventions are indeed complex and heavily skill-dependent procedures, subject to improvements in technical performance. Quality in performance usually requires extensive training over time–a ‘learning curve’, which is difficult to control in a clinical trial. In fact, during the learning curve phase, errors are likely to occur, and this could greatly distort the outcome results.

Difficult as these problems may be, there are, nonetheless, some approaches that would make surgical trials feasible [9, 10]. Learning curves and variations in techniques can be measured and controlled as well [11]. For example, one strategy to account for learning curves is to design a pilot trial, or a run-in period, that will be discounted in the final analysis. Another strategy is to prespecify in the analytic plan of the study that investigators will explore whether significant changes occur in outcomes throughout the trial. This would allow the researchers to assess whether a learning curve effect exists, and to control for it. Moreover, to guarantee the standardization of the procedure, participation may be limited only to surgeons with particular skill levels. This can be done, for instance, by conducting the trial in highly specialized centers with specific surgical expertise. Another promising idea to standardize surgical interventions comes from Blencowe and colleagues [12], who developed a typology of surgical interventions for use during trials design. This framework aims at describing surgical interventions in sufficient detail to enable the accurate replication of the procedure over the trial. This approach has been successfully adopted in two surgical RCTs to identify the key intervention components and steps needed to be standardized, as well as the degree of standardization required for each [12].

### Blinding

The second methodological challenge for conducting RCTs in surgery is the blinding issue. Blinding and placebos are particularly difficult to apply in the real world: surgery cannot be blinded in any way, whereas the adoption of sham procedures as controls raises several ethical concerns. Without proper blinding, biases can contaminate the trial outcome, making the experiment scientifically flawed. Patients are also reluctant to undergo treatment randomization in surgery. The procedures under testing are often perceived as highly unequal, regarding the invasiveness of the intervention, side effects, or quality of life. Hence, patient enrollment becomes difficult, making trials difficult to complete for lack of a proper sample size. In this regard, however, a recent systematic review reports that randomized surgical trials are feasible, that placebo and blinding can be easily implemented, and that drop-out rates are very low [13]. Nonetheless, patient recruitment is usually quite slow. There is an assumption that patients in severe pains are unwilling to take part in surgical RCTs, but surprisingly, patients who were suffering more were more likely to agree to take part in the trial. Why eligible patients are reluctant to participate in trials remains to be understood. But still, this does not seem like an insurmountable barrier. Of course, double-blinded placebo-controlled trials are nearly impossible unless the study intervention is a medication or a transplant. Nonetheless, the single-blind design should be sufficient to control at least for some potential bias, to the extent to which the surgeon’s role is limited to the surgery, and the evaluation of benefits, data analysis, and patient’s care is provided by blinded actors.

To summarize, difficult as these methodological problems may be, and although we cannot expect surgical trials to reach the same level of dependability of pharmaceutical RCTs, some solutions would make surgical trials feasible and scientifically sound. Critics might argue that, despite these solutions, we are still far from the level of treatment standardization that we should have in ideal RCTs. This is certainly true, but we must acknowledge that, in certain cases, possibilities exist to implement at least some approximations of RCTs. Moreover, it should be emphasized that the level of such approximation is highly dependent on the nature of the procedure under testing. According to the least optimistic review [14], in principle, well-designed RCTs can be performed to evaluate 40% of treatment questions involving surgical procedures–and the authors consider it a conservative estimate.

### Pragmatic concerns

On the pragmatic side, there are two major obstacles to the conduct of surgical trials. On the one hand, there is a funding issue: clinical trials are very expensive experiments and, unlike with pharmacological treatments, there is no clear sponsor for surgical tests. Publicly funded surgical trials are not a priority for any political party, as of today, and, even if they were, the costs would not be sustainable for any western democracy. Nonetheless, in public healthcare systems, it is crucial to carefully evaluate the effectiveness of treatments, to make an informed cost-benefit analysis that would guide the allocation of limited healthcare resources. To do this, any cost-effectiveness analysis for a good and fair allocation of resources needs to be based on the best possible information about the net effects of treatments. Even third parties, such as insurance companies or hospitals, might have a strong financial interest in running surgical trials (e.g., for removing expensive and inefficient procedures from their portfolios).

Second, surgical interventions are considered–at least in the US–a medical *practice*, and as such, they are outside the scope of any dedicated regulatory body: the FDA can only examine products, not services. In the 1960 and 1970 s, the US medical profession resisted state intervention in medical practice (e.g., prescription assignment) as a form of communism [15]. For the same reasons, the FDA does not regulate behavioral and psychological interventions, nor the use of off-label drugs. These ideas, however, are based on questionable political assumptions. Also, despite the prestige of the surgical profession, it is hard to believe that the community of surgeons is powerful enough to resist the external imposition of more demanding testing standards when the omnipotent pharmaceutical companies are so heavily regulated.

## Ethical concerns in surgical RCTs

A third battery of arguments against surgical trials comes from bioethics. Many scholars have pointed out several ethical issues that make placebo-controlled surgical trials morally unacceptable [16]. One of the most discussed problems is the use of sham procedures as controls. To be methodologically sound and scientifically reliable as pharmaceutical RCTs, surgical trials require the use of ‘surgical placebos’, which consist in sham procedures–e.g., mere skin incisions or endoscopic techniques with no direct intervention on anatomy or physiology, and no intended beneficial therapeutic effect–whose function is just to simulate the actual surgery. This way, psychological effects (e.g., expectations) would be comparable in both arms of the trial, potential placebo effects would be controlled, and biases reduced.

Unsurprisingly, the use of such ‘fake’ procedures has led surgeons and bioethicists to judge placebo-controlled surgical trials as unethical. In fact, sham procedures–unlike chemically inert placebo pills–carry the same risks of real surgical treatments (such as infections, hernia, blood loss) as well as risks associated with related pharmaceutical treatments (such as anesthesia and/or antibiotics). For this reason, “using a sham surgery component in the control […] adds risks of foreseeable and preventable harm without a corresponding benefit to subjects in the control arm” [17], and this, according to several scholars, makes placebo-controlled surgical trials immoral.

However, as Franklin Miller has suggested [18, 19], such negative attitudes, in the bioethical debate, towards placebo-controlled surgical trials are likely to depend on a biased, partial analysis of the problem. In fact, the ethical discussion has mainly focused on quite extreme cases, which are undoubtedly very problematic–if not patently unacceptable–from an ethical standpoint. For instance, one of the most cited, discussed, and criticized examples of sham surgery as control in a trial is a case of fetal cell implantation in the brain of Parkinson’s patients–an experiment conducted in the 1990s [20]. This study involved the transplantation of aborted human fetal tissue through scalp incisions and drilling holes in the skull of patients; in addition to these very invasive and risky procedures, there was also a high risk of complications and side-effects associated with related immunosuppressive treatments, antibiotics, and PET scan radiations. In cases like this, it is hard to disagree that harms and risks are disproportionate to the expected benefits of the trial [16].

But there are also less controversial cases of sham-controlled trials in surgery, where a more positive risk-benefit ratio makes them less morally problematic. For instance, another largely discussed case in the bioethical literature is a trial conducted to assess the effectiveness of the ligation of the internal mammary artery for the treatment of angina. This practice was a very common procedure in the 1950s [21] until two sham-controlled trials revealed that it was no better than a sham intervention consisting of a simple skin incision under local anesthesia, without involving the critical surgical element of the operation. In light of these results, the procedure was abandoned. Bioethicist Henry Beecher applauded this conclusion in his seminal paper “Surgery as Placebo” [22], emphasizing how sham-controlled trials had shown their great potential to inform surgical practice and to secure future patients from risky and ineffective operations.

A more recent, emblematic randomized placebo-controlled clinical trial tested arthroscopic surgery for knee osteoarthritis [23]. This is still a very common procedure but, like many others, its efficacy had never been assessed before introducing it into clinical practice. Before running the trial, this practice was performed hundreds of thousands of times per year in the US, costing approximately 5.000 dollars per intervention. The trial results showed that this surgery was no better than a sham operation, suggesting that thousands of patients had been (and continue to be) exposed to the risks of a costly and ineffective procedure with almost no direct benefit. As Beecher said, these results should suggest we “question the moral or ethical right to continue with casual or unplanned new surgical procedures–procedures which may encompass no more than a placebo effect–when these procedures are costly of time and money and dangerous to health or life” [22].

As this evidence suggests, surgical trials appear not only able to satisfy some minimal methodological standards to make them even just approximately as dependable as drug-RCTs; they can also be morally justified, even if they adopt sham procedures as control.^2^ The moral acceptability of placebo-controlled RCTs in surgery is a very important criterion to evaluate the overall feasibility of these trials. Assessing the actual overall possibility of running RCTs–in light of the epistemological, pragmatic, and ethical concerns that they trigger–is a necessary task if we believe that better evidence about surgical treatments’ safety and efficacy is needed, and that a stronger public regulation should be implemented. Practical philosophers call the general principle behind this idea ‘ought implies can’: claiming that something should be done makes no sense if it is empirically impossible to do it.

As already stressed, our claim is not that *every* kind of surgical procedure should be tested through RCTs, since not any possible kind of placebo-controlled surgery satisfies the necessary conditions for a methodologically sound and ethically justified trial. In the following section, we consider some conditions that need to be met to run morally acceptable RCTs.

### Conditions for the moral acceptability of surgical RCTs

As we have seen, ethical considerations are decisive to decide whether a trial testing a surgical intervention against a sham procedure is morally acceptable or not. Such evaluation depends on complex risk-benefit analyses involving scientific evidence, statistical considerations, reports, and value judgments as inputs.

Since relevant reports and data are usually relative to specific surgical procedures, risk-benefit considerations should be relative to specific interventions. Even very different net risk-benefit ratios can result from the analysis of hypothetical trials involving different surgical procedures. Some of them could, therefore, be more morally acceptable than others. This implies, once again, that it is not possible to run RCTs for every kind of surgical intervention. But this is not a sufficient reason for testing no surgical procedure at all.

Drawing on the cases discussed above and following a recent ethical framework suggesting guidelines for surgical trials [24], we can identify some conditions to be fulfilled for conducting morally acceptable RCTs.

### Clinical equipoise

The concept of clinical equipoise refers to the presence of genuine uncertainty about the efficacy of treatment and/or its superiority or inferiority as compared to other treatments for the same condition. It is considered one of the most important principles in the ethics of clinical research, and it was introduced as a criterion to respect both the principles of beneficence and justice included in the Declaration of Helsinki. Although it requires a genuine uncertainty about the risk-benefit ratio of a certain treatment, the criterion of equipoise also requires the presence of sufficient preliminary evidence about the efficacy and safety of the treatment at stake; such preliminary evidence can be, for instance, the result of earlier trials or studies conducted on non-human animals. However, the principle of equipoise also requires that there not be an already stated rigorous scientific demonstration of these outputs, which is precisely what the research through RCTs aims to provide. This criterion can be conceived, on the one hand, as an instantiation of the principle of beneficence, since its requirement of preliminary evidence aims to protect the patients from inappropriate risks during and after the research. On the other hand, the criterion of clinical equipoise also aims to respect the principle of justice, since all patients should have an equal chance to receive the best available treatment in terms of safety and efficacy. Therefore, clinical equipoise is considered a necessary condition to start any RCT.

### Minimization of risks

A second, important condition concerns the minimization of risks for patients, which can be seen as an instantiation of the Declaration of Helsinki’s principle of non-maleficence. According to this principle, patients should not be exposed to serious and irreversible harms when enrolled in clinical research. This should suggest a special concern for patients in the placebo arm, which receive no direct benefit from the tested treatment (other than potential placebo effects). For instance, in the case of the aforementioned research on fetal cells transplantation to Parkinson patients, there was no effective therapy at all at the time, so the principle of clinical equipoise was respected. Nonetheless, the risk for patients was too high and disproportionate as compared to the expected benefits (perhaps, even for patients in the experimental arm). But as far as other kinds of procedures are concerned, the risk-benefit ratio–which, once again, is always highly contextual–can change. Notice that, in the few published placebo-controlled trials in surgery, the occurrence of harms or adverse events which might be associated with the sham procedures–side-effects which can occur in almost every kind of surgery, such as blood loss or infection–is nearly never mentioned. This suggests that, in many cases, the risks associated with certain sham procedures are almost negligible.

### Direct benefit

A further condition concerns the presence of at least some direct benefit to patients in the placebo arm, other than the placebo effect. First, sham surgery should deliver some sort of benefit to patients in the placebo arm: for instance, providing them with new diagnostic information. Second, the placebo arm should not be left without any treatment: this may consist in the addition of an effective treatment during the trial (to both arms). Such a treatment may be pharmaceutical or not: for instance, many surgical patients receive specific rehabilitation therapy after the intervention. So, patients receiving placebo may still benefit from it.

In general, the moral importance to satisfy these conditions, combined with the specificity of the risk associated with each surgical procedure, and differences in the availability of objective outcome-measures to reliably evaluate the impact of surgical interventions suggests that RCTs cannot be acceptable (or so urgently needed) for every kind of surgery, but rather that “placebo-controlled trials in surgery will most often be justified when surgery is performed to improve function or relieve symptoms, and when objective outcomes are not available” [24]. This also means that trials would be unethical when sham surgery implies delaying an actual treatment to some patients, and when this delay could result in severe complications in the pathological condition. However, in many cases, delaying critical surgical procedures–which are aimed, for instance, at improving subjective life quality or at reducing self-reported pain–does not necessarily produce bad outcomes. The placebo arm can even, in some cases, benefit more than the control arm (e.g., avoiding exposing themselves to the risks related to the critical surgical element of an ineffective procedure). This can subsequently lead to providing patients in the placebo arm with another, safer, and more effective treatment after the trial (assuming there is one).

Finally, we address a potential objection to our view. Even if surgical RCTs are feasible and morally justified, some might claim that higher standards of evidence seem *irrelevant* for many surgical procedures. Surgical treatments are indeed a very heterogeneous category, ranging from tooth extraction to awake brain surgery, which largely differs in terms of complexity and rationale. Among surgical treatments, there are life-saving procedures, whose efficacy is often unquestionable. In other words, some medical interventions are so evidently beneficial that it would be meaningless (impractical and unreasonably expensive) to test them with RCTs. To illustrate this point, some scholars [25] likened these practices to the use of a parachute during a free fall from an airplane. Without the use of a parachute, the risk of death approaches nearly 1, while with a parachute it decreases dramatically to nearly 0. Some surgical procedures can be analogous to a parachute, and we do not need a clinical trial to test them. For example, sewing a wound up works great if someone is hemorrhaging. However reasonable, this analogy has its limitations. Parachute-like treatments are very rare in medicine [26] and the efficacy of practices that have been likened to a parachute have recently been shown to be significantly overstated [27]. So, even in surgery, except for a handful of procedures–which are, for instance, normally applied in emergency contexts–we maintain that there are good reasons to believe that we would greatly benefit from better evidence about the efficacy of surgical procedures, as long as the basic conditions to obtain such evidence can be respected.

## Surgical regulation

The current testing procedure of surgical treatments, on its own, seems difficult to defend. The vast majority of surgical procedures are regularly performed without any statistical testing, relying mostly on experts’ judgment, anecdotal evidence, or case series. But these assessments often fail, and–since surgical interventions are rarely riskless–several procedures introduced in this way into clinical practice end up exposing patients to unnecessary harms. The history of medicine is littered with interventions, including surgical procedures, that were believed effective and safe but that fell apart with randomized trials [29].^3^

So far, we have analyzed some of the main reasons why, at present, surgery is not regulated in the same way as drugs are. All these reasons emphasize problems–methodological, pragmatic, and ethical–related to the specific procedures through which scientific evidence about surgical interventions might be collected. Nonetheless, we have offered arguments and evidence against these reasons and shown that, under certain conditions, surgical trials can be methodologically sound and morally acceptable. But why should we need them? Why do we think regulating surgical procedures–by testing them with RCTs when possible–is a good thing? Would the institution of a regulatory organization (such as the FDA) which constrains the liberty of receiving (for patients) and introducing or performing (for surgeons) any kind of surgical procedure be a justified public interference with individual autonomy?

Intuitively, it seems reasonable to believe that surgical patients deserve a level of protection, evidence, and information that at least approximates–when possible–that of pharmaceutical consumers. We claim that a more dependable evaluation of surgical interventions could best serve patients, even just by providing them with better information about the potential risks they might run when making a therapeutic choice. Whether it concerns a pharmaceutical or a surgical treatment, a therapeutic choice should be informed as much as possible: this is the best way to avoid distorting influences, such as the economic interest of other stakeholders (e.g., surgeons, clinics, insurance companies) and strong paternalistic interferences. However, reliable and transparent information about risks and benefits of the available therapeutic options would be unlikely to be collected or given by their direct providers in a free, unregulated market. Furthermore, it is practically impossible to obtain by patients alone, merely through observation and experience. The best way to dispose of a reliable, disinterested, impartial evaluation of the efficacy and safety of medical treatments is to have it provided by independent regulatory bodies, such as the FDA [30].

But this requires, as for drugs, some therapeutic options to be at least temporarily prohibited until they pass a rigorous, independent evaluation of efficacy and safety. Just as drug consumers are not able to reliably assess the risks and benefits of pharmaceutical treatments by themselves, and as market and drug companies are unlikely to provide transparent and unbiased information, a proportional logic should apply to surgical patients who often accept to receive very risky treatments (often even riskier than drugs) which have never been rigorously tested.^4^ This moderately ‘paternalistic’ stance, consisting in constraining to perform or receive surgical treatments “without the consent of neither physicians nor patients, for the sake of their good health” [30] is based on the acknowledgment of clear information asymmetry. It is what Rebecca Dresser has called a “Ulysses contract” [31]: since patients alone are unable to ascertain the risks related to treatment, it is rational for them to accept to be constrained, by an independent regulatory agency, in their freedom to access them, waiting for a verdict on their efficacy and safety [32]. In this way, regulatory agencies can provide patients with full disclosure about their risks, allowing them to make more informed therapeutic decisions.

But what could surgical regulation look like? Concerning the introduction of new procedures, the reply appears quite similar to the justification of certain limits on the introduction of new drugs on the market. In this case, the constraint to patients’ and surgeons’ liberty consists in a temporary ban to perform and to access the new surgical procedures until tested in RCTs. So, we might envisage an FDA-like regulatory body, requiring surgeons to show that a new surgical procedure is safe and effective before introducing it in the clinical routine. As far as widespread and commonly performed procedures are concerned, however, things get much more complicated. It would be, of course, unthinkable and undesirable to ban all currently available surgical procedures until tested with RCTs (also because, as said, very likely RCTs testing might not be possible for all surgical procedures). And, even if that were possible, we could not test them all overnight. So, what could we do? In this regard, the regulation of medical devices can provide useful insights.

Since 1976, the FDA has also handled a regulatory mandate for medical devices. In that year, FDA officers were suddenly tasked with sifting through a huge volume of “medical products” falling within FDA jurisdiction. With scarce resources, they had to prioritize their efforts. Therefore, the FDA decided to assign categories of risk associated with devices, grounding on both background knowledge and common sense. Intuitively, some medical products seemed less risky than others: latex gloves carry less risk than cardiac pacemakers, even if both count as devices for regulatory purposes. Hence, the agency created three different device classes (I, II, III), according to the risks that they can pose to patients. In light of this distinction, the FDA requires pre-marketing testing and approval only for high-risk devices (Class III). While medical device regulation has not been particularly successful in protecting device consumers^5^, it may nonetheless pave the way for a regulatory framework of existing surgical treatments. Based on current knowledge and observational data, we could identify high-risk surgical procedures and require RCTs to check for their efficacy and general safety, assuming that the necessary ethical conditions outlined in earlier sections are met. If some surgical procedures turn out to be unsafe or ineffective, then they should be prohibited. Moreover, a regulatory authority for surgery should mandate continuous monitoring of surgical procedures to collect data and identify those procedures whose safety and efficacy are uncertain and require clinical trials.

## Conclusions

Due to the lack of robust statistical evidence, surgeons often make critical treatment decisions based only on their clinical judgment and professional experience, and patients are not provided with reliable information about the outcomes of interventions. This is because surgical procedures are left completely unregulated and can be introduced into clinics nearly without any scientific evidence. On the contrary, pharmaceutical treatments are heavily regulated by governmental agencies, such as the FDA, that exercise gatekeeping power requiring two positive RCTs for granting market access to new drugs. Several arguments can explain the lack of surgical regulation. Most of them concern the feasibility of RCTs to test surgical procedures. We have reviewed these arguments, suggested that they are not decisive, and concluded that there is room for conducting scientifically sound and ethically acceptable trials even in surgery. Setting up a more robust regulation for surgical treatments would improve medical decision-making and would provide patients with more reliable information about risks and benefits associated with surgical procedures, protecting them from ineffective, risky, and costly interventions.

## Data Availability

Not applicable

## Footnotes

1 We are aware that many scholars would disagree with this claim. There has indeed been criticism of the FDA for having set the bar too low and some have called for raising it.

2 For a more extensive discussion of the aforementioned cases, see the ethical analysis conducted by Miller [18].

3 A well-known example is the percutaneous coronary intervention to reduce the risk of death or nonfatal myocardial infarction in patients with stable coronary disease, refuted by the COURAGE trial [28].

4 Some may object that a reduction in the number of therapeutic options proposed by surgeons to patients because of a lack of evidence might affect the surgeon-patient relationship. We disagree on this point. We believe it is better to propose fewer options, whose effectiveness and safety have been rigorously assessed, rather than to offer therapies whose risk-benefit ratio is unknown.

5 See e.g., https://www.theguardian.com/society/2018/nov/25/revealed-faulty-medical-implants-harm-patients-around-world [Online: Accessed 18/06/2021].
